# Hypertensive disorders in pregnancy and child development at 36 months in the All Our Families prospective cohort study

**DOI:** 10.1371/journal.pone.0260590

**Published:** 2021-12-01

**Authors:** Natalie V. Scime, Erin Hetherington, Lianne Tomfohr-Madsen, Alberto Nettel-Aguirre, Kathleen H. Chaput, Suzanne C. Tough

**Affiliations:** 1 Department of Community Health Sciences, University of Calgary, Calgary, Alberta, Canada; 2 Department of Obstetrics and Gynaecology, University of Calgary, Calgary, Alberta, Canada; 3 Department of Psychology, University of Calgary, Calgary, Alberta, Canada; 4 Department of Pediatrics, University of Calgary, Calgary, Alberta, Canada; 5 Centre for Health and Social Analytics, NIASRA, School of Mathematics and Applied Statistics, University of Wollongong, Wollongong, New South Wales, Australia; University of Oslo, NORWAY

## Abstract

Hypertensive disorders in pregnancy (HDP) are associated with increased risk of offspring neurodevelopmental disorders, suggesting long-term adverse impacts on fetal brain development. However, the relationship between HDP and deficits in general child development is unclear. Our objective was to assess the association between HDP and motor and cognitive developmental delay in children at 36 months of age. We analyzed data from the All Our Families community-based cohort study (n = 1554). Diagnosis of HDP–gestational or chronic hypertension, preeclampsia, or eclampsia–was measured through medical records. Child development was measured by maternal-report on five domains of the Ages and Stages Questionnaire (ASQ-3). Standardized cut-off scores were used to operationalize binary variables for any delay, motor delay, and cognitive delay. We calculated adjusted risk ratios (aRRs) and 95% confidence intervals (CIs) using logistic regression, sequentially controlling for potential confounders followed by factors suspected to lie on the causal pathway. Overall, 8.0% of women had HDP and hypertension-exposed children had higher prevalence of delay than unexposed children. Hypertension-exposed children had elevated risk for developmental delay, but CIs crossed the null. The aRRs quantifying the fully adjusted effect of HDP on child development were 1.19 (95% CI 0.92, 1.53) for any delay, 1.18 (95% CI 0.86, 1.61) for motor delay, and 1.24 (95% CI 0.83, 1.85) for cognitive delay. We did not find a statistically significant association between HDP and developmental delay. Confidence intervals suggest that children exposed to HDP in utero have either similar or slightly elevated risk of any, motor, and cognitive delay at 36 months after controlling for maternal and obstetric characteristics. The observed direction of association aligns with evidence of biological mechanisms whereby hypertensive pathology can disrupt fetal neurodevelopment; however, more evidence is needed. Findings may have implications for early developmental monitoring and intervention following prenatal hypertension exposure.

## Introduction

Hypertensive disorders of pregnancy (HDP) are common medical complications affecting up to 9% of women, and include gestational hypertension, pre-eclampsia, and eclampsia [1]. In addition to negatively impacting maternal and fetal health and well-being [2–7], HDP also appear to have long-term neurodevelopmental impacts for children. A meta-analysis of 61 studies concluded that neurodevelopmental disorders, such as autism and attention-deficit/hyperactivity disorder (ADHD), occur roughly 30% more frequently in children and adults exposed to HDP in utero [8]. Hypertension is accompanied by reductions in placental blood flow, oxygenation, and nutrient transfer that are thought to disrupt the highly sensitive process of fetal brain development [9]. The potential mediating role of preterm birth has been raised [10, 11]; HDP is resolved through medically indicated delivery and early delivery impacts neurodevelopment. However, research on children born at term have found similarly elevated risk of cerebral palsy, autism, ADHD, epilepsy, and intellectual disability in those exposed to preeclampsia, suggesting a direct link between HDP and neurodevelopment independent of gestational age at birth [12].

In addition to clinical diagnoses, a number of studies have targeted the relationship between HDP and child development. Studies examining early childhood (i.e., 6–36 months) have generally analyzed small samples of preterm, growth restricted, or small-for-gestational age infants, and have reported a mix of negative [13, 14], positive [15, 16], or null [17, 18] associations between HDP and offspring cognitive scores. Studies on motor abilities at 18–24 months have reported conflicting results, with four studies on preterm or small-for-gestational age infants finding no differences in motor function according to in utero HDP exposure [14, 16–18], and one study on infants of any gestational age finding gross motor delay to be significantly more common among hypertension-exposed (46.2%) than unexposed offspring (5.3%) [19]. By contrast, research using large birth cohorts have linked HDP to lower mean verbal ability scores and increased odds of mild cognitive limitations at 10–11 years of age [20, 21], as well as lower mean composite motor scores at 10 and 14 years [22].

Existing evidence supports that HDP heightens the risk of neurodevelopmental diagnoses in offspring, and that a relationship between HDP and deficits in cognitive and motor development may exist but is less consistently observed. Studies targeting general child development are of lower quality due to variations in sampling frame (i.e., targeted to preterm or small babies over the general population) and small study sizes (n<250), which likely contributes to variation in results. Moreover, the association between HDP and child development is often studied in the context of developmental *scores*, which fails to address whether differences in mean group abilities translates into meaningful differences in function based on validated thresholds of normal versus delayed development. Thresholds are generally used over scores in practice settings to identify children experiencing issues relative to population norms. Therefore, the objective of this study was to assess the association between HDP and motor and cognitive developmental delay in children at 36 months of age.

## Methods

### Study sample

We performed a secondary analysis of data from the All Our Families (AOF) community-based pregnancy cohort study comprised of 3,388 families in Calgary, Canada and surrounding areas. Details about the AOF cohort design are outlined elsewhere [23]. In brief, women aged 18 years or older who were less than 25 weeks pregnant were recruited through primary care offices, public health laboratory services, and community posters from May 2008 to December 2010. The AOF database includes extensive information on demographics, lifestyle, mental health, health service use, and child development with data collected through mother-reported questionnaires during pregnancy (22–24 and 32–36 weeks), the postpartum (4- and 12-months), and childhood (2-, 3-, and 5-years), as well as medical record data on labour and delivery linked via personal health numbers.

The sample for this analysis included mother-child dyads who responded to the 3-year questionnaire and consented to record linkage. Dyads were excluded if the child development portion of the questionnaire was missing or if the child’s age was outside of the assessment window (34 months/16 days to 38 months/30 days). We also excluded children with a diagnosed neurodevelopmental disorder reported within the first 3 years, to maintain a focus on general child development and not neuropathology.

### Exposure

HDP included chronic hypertension, gestational hypertension, preeclampsia, and eclampsia [24]. Disorders were documented by birthing providers on delivery records using diagnostic codes from the International Classification of Diseases 10^th^ Revision (Version: 2008, Canada). Chronic hypertension was defined as hypertension (systolic blood pressure ≥140 mmHg or diastolic blood pressure ≥90 mmHg) with onset predating pregnancy, and was measured with code O10. Gestational hypertension was defined as new-onset hypertension after 20 weeks gestation, and was measured with code O13. Preeclampsia is a more severe, multi-system syndrome defined as gestational hypertension with proteinuria (i.e., excess urinary protein), and was measured with code O14. Eclampsia is an extension of preeclampsia involving maternal seizures, and was measured with code O15.

### Outcome

Child development at 36 months was measured by maternal report on the Ages and Stages Questionnaire third edition (ASQ-3) targeting five domains: communication, problem solving, personal-social (together comprising cognitive development), gross motor, and fine motor (together comprising motor development) [25]. The ASQ-3 has good psychometric properties [26] and has been widely adopted for community-based developmental screening. Each domain contains six questions with responses of ‘yes’ (10 points), ‘sometimes’ (5 points), or ‘not yet’ (0 points). Responses are summed into a domain score ranging from 0 to 60, and higher scores indicate better development. ASQ-3 cut-offs for the ‘monitoring zone’ (score between 1 and 2 standard deviations [SD] below the mean) and ‘referral zone’ (score ≤2 SD below the mean) were established in a normative sample of 18,572 American children representative of the ethnic and socioeconomic distributions in the population [25], and have been externally validated against professionally administered standardized assessments [27, 28]. We operationalized developmental delay as showing monitoring or referral scores (≤1 SD below the normative mean) for at least one of five domains (any delay), at least one of three cognitive domains (cognitive delay), and at least one of two motor domains (motor delay).

### Covariates

Covariates were chosen based on a literature review and dataset availability, and included maternal age at baseline (numeric, years), sociodemographic vulnerability (yes/no), pre-pregnancy overweight or obesity (body mass index ≥30/<30), parity (nulliparous/multiparous), depression during pregnancy (yes/no), depression at 4 months postpartum (yes/no), child sex (male/female), and gestational age at birth (numeric, completed weeks). Sociodemographic vulnerability was operationalized using a composite indicator [29], with ‘vulnerability’ defined as ≥2 of: annual household income <$60,000 (the eligibility threshold for subsidized housing at the time of data collection), maternal education of some post-secondary or less, single marital status (including divorced, separated), does not own current home, and primarily speaks a language other than English at home. Maternal depression was measured using a cut-point score of ≥10 on the Edinburgh Postnatal Depression Scale, which is appropriate for a community sample [30, 31].

### Statistical analysis

**Fig 1:**
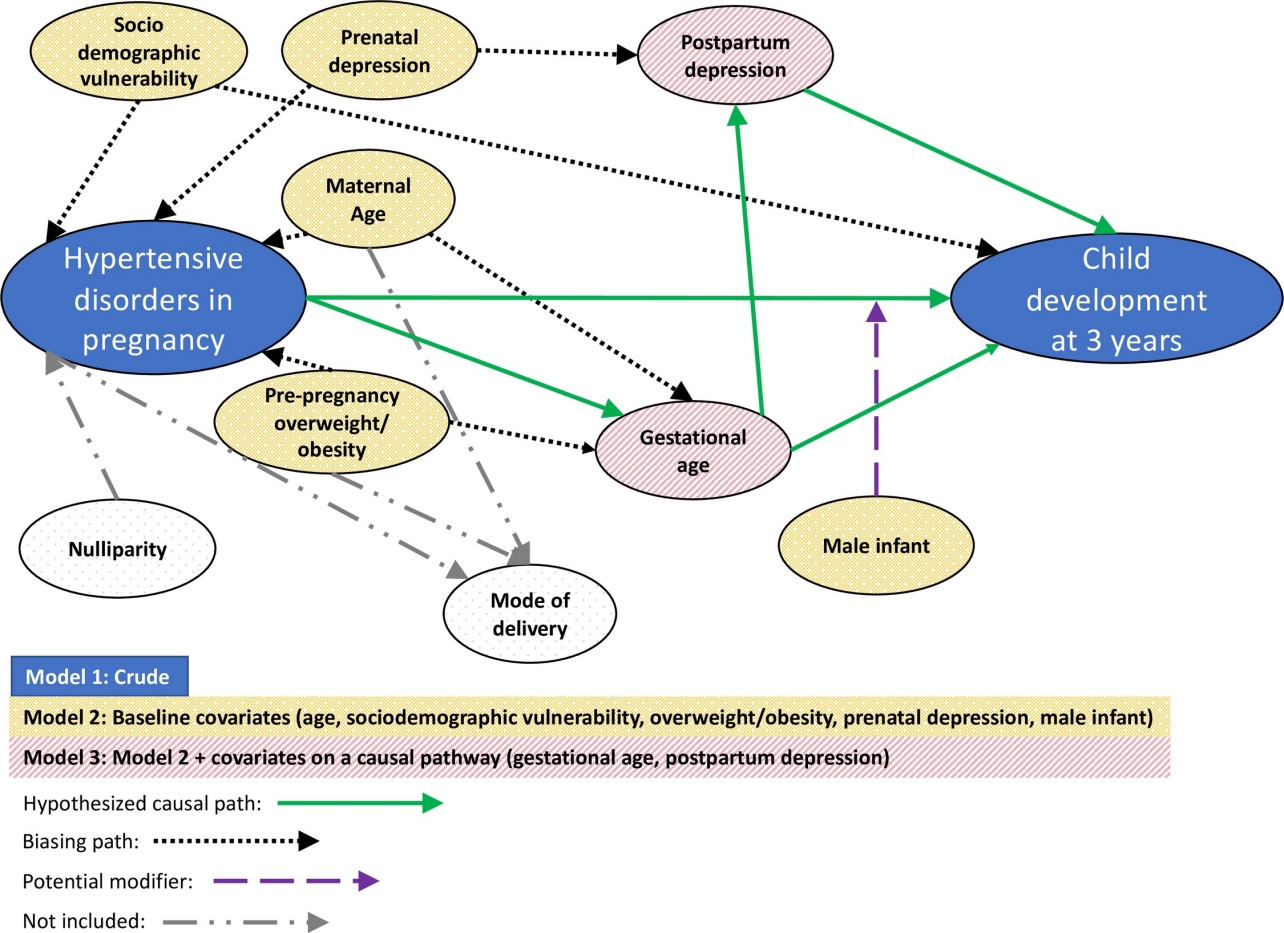
Directed acyclic graph depicting the relationship between hypertensive disorders in pregnancy and child development and relevant covariates available for this analysis.

Descriptive statistics were used to explore and summarize the data. We constructed multivariable logistic regression models followed by the adjrr command in STATA to estimate RRs and 95% CIs for developmental delay categories [32], comparing children exposed to HDP to those who were not. We developed a directed acyclic graph (DAG; see Fig 1) to document our assumptions about the relationship between the covariates, exposure, and outcome, and specifically to hypothesize which variables were confounders (preceding both exposure and outcome) and which were factors suspected to lie on the causal pathway. This DAG informed our staged modelling approach: [33] model 1 was crude (unadjusted), model 2 was adjusted for confounders (sociodemographic vulnerability, maternal age, pre-pregnancy overweight/obesity, prenatal depression), and model 3 was adjusted for confounders and factors on the causal pathway (gestational age, postpartum depression). Child sex was assessed as a potential modifier using interaction terms; in the absence of evidence for modification, sex was included in adjusted models to maintain consistency with published literature. Parity and mode of delivery were excluded because they were not hypothesized to be on a pathway affecting the exposure-outcome relationship. Continuous covariates were modelled using restricted cubic splines with 4 knots at the 5^th^, 35^th^, 65^th^, and 95^th^ percentiles of the data to allow for flexible and non-linear distribution [34]. We conducted two sensitivity analyses. First, we repeated our main analysis restricting our definition of HDP to cases of pre-eclampsia and eclampsia, to examine whether associations differed when narrowing focus to the most severe cases of HDP. Second, we repeated our main analysis restricting to children born at full-term (37 weeks or greater), to examine whether adjusting for gestational age in our main models may have inadvertently introduced bias through unmeasured confounding between gestational age and child development [35].

Information on missing data is outlined in S1 Table. We handled missing covariate data (9.4% of participants) using complete case analysis and sample sizes for each model were reported. We used a conservative approach for classifying developmental status of children with data missing on one domain (1.5%); we were unable to classify overall development for 15 children, motor development for 7 children, and cognitive delay for 12 children.

The AOF study (REB15-0852 and REB13-0868) and this secondary analysis (REB18-1779) received ethics approval from the Conjoint Health Research Ethics Board at the University of Calgary.

## Results

**Fig 2:**
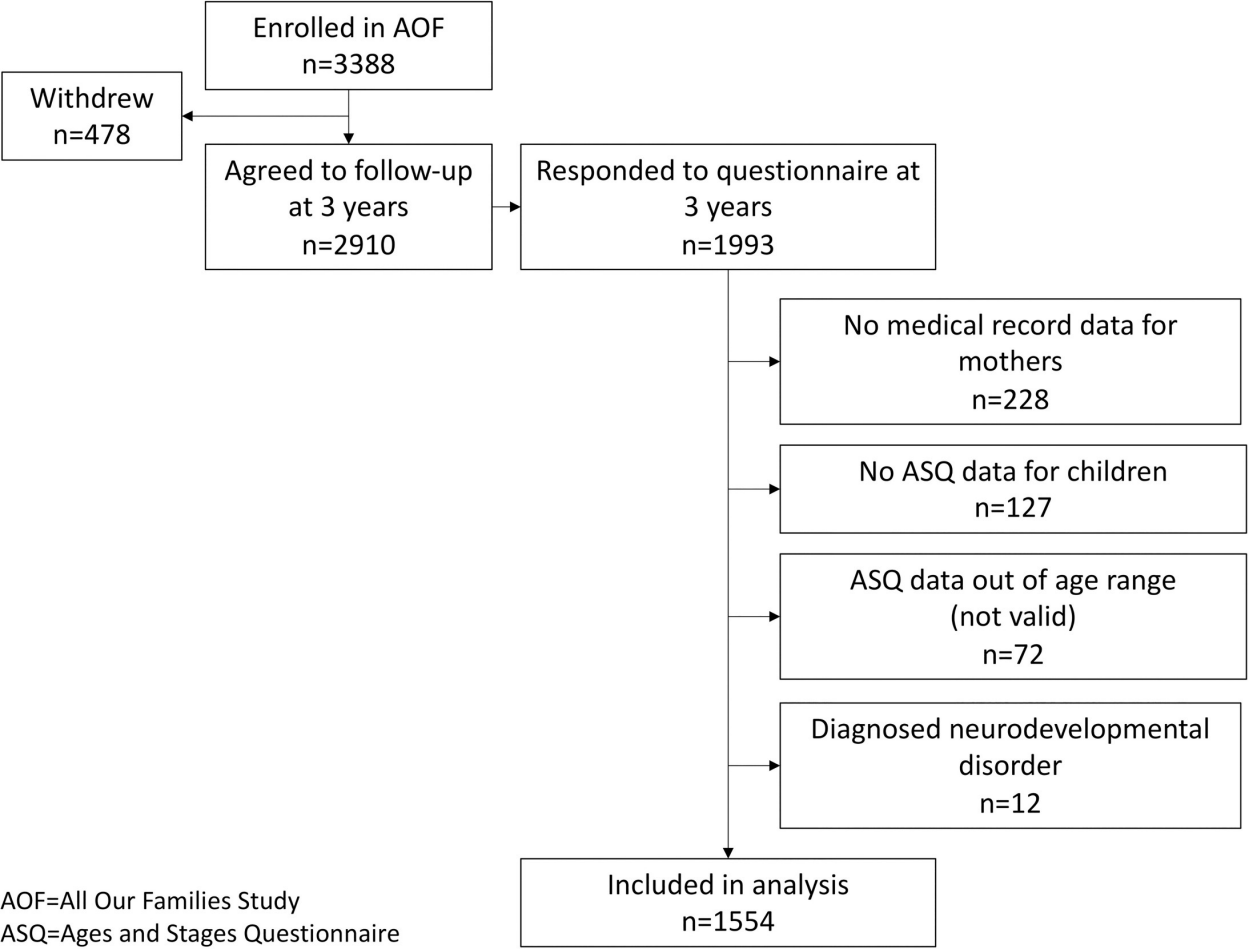
Flowchart of study participants.

Sample selection for this analysis is depicted in Fig 2. Of the 2909 women still enrolled in the AOF cohort at 3 years, 1993 completed the 3-year questionnaire (68.5% response rate). After excluding those with missing hypertension data, missing or invalid child development data, or a neurodevelopmental disorder in the child, 1554 mother-child dyads were included. Our analytic sample was fairly representative of the full 3388 participants in the AOF cohort (see S2 Table).

Sample characteristics overall and stratified by hypertensive status are displayed in Table 1. The prevalence of HDP was 8.0%, with the majority of these 124 women experiencing gestational hypertension (83.9%) or preeclampsia (75.0%). A greater proportion of women with HDP were overweight or obese, nulliparous, and experienced prenatal depression compared to non-hypertensive women. The proportions of women experiencing sociodemographic vulnerability and postpartum depression were similar across exposure groups. Women with HDP were more likely to have had a cesarean birth and delivered a preterm infant.

**Table 1:** Sample characteristics.

	Overall	Hypertensive Status	
Characteristic	N = 1554 n (%)	HDP+	HDP-
		N = 124 n (%)	N = 1430 n (%)
Hypertension type			
Chronic hypertension	--	8 (6.5)	--
Gestational hypertension	--	104 (83.9)	--
Preeclampsia	--	93 (75.0)	--
Eclampsia	--	17 (13.7)	--
Maternal age at delivery, mean (SD)	31.4 (4.4)	31.9 (4.5)	31.3 (4.4)
Sociodemographic vulnerability	235 (15.3)	17 (14.1)	218 (15.4)
Single	19 (1.2)	2 (1.6)	17 (1.2)
Does not own home	274 (17.6)	21 (16.9)	253 (17.7)
Primarily speaks non-English language	152 (9.8)	11 (8.9)	141 (9.9)
Annual income <$60,000	200 (13.4)	17 (14.3)	183 (13.3)
Some post-secondary education or less	310 (20.0)	26 (21.0)	284 (19.9)
Pre-pregnancy overweight or obesity	522 (34.0)	84 (68.9)	438 (31.0)
Primiparous	780 (50.6)	87 (71.3)	693 (48.8)
Perinatal mental health			
Prenatal depression	329 (21.4)	36 (29.5)	293 (20.7)
Postpartum depression	176 (11.6)	15 (12.3)	161 (11.5)
Cesarean delivery	380 (24.9)	45 (36.6)	335 (23.8)
Gestational age at birth (mean [SD])	39.0 (1.7)	37.8 (2.3)	39.1 (1.6)
Preterm birth	89 (5.7)	24 (19.4)	65 (4.6)
Child male sex	825 (53.1)	63 (50.8)	762 (53.2)

HDP = hypertensive disorders in pregnancy. There is some variation in the denominator used for each characteristic due to missing data; the denominator included only participants who provided data for each characteristic.

Prevalence estimates for developmental delays in children at 36-months overall and stratified by in utero hypertension exposure are shown in Table 2. Overall, the prevalence any delay, motor delay, and cognitive delay were 32.0% (95% CI 29.7, 34.4), 23.5% (95% CI 21.5, 25.7), and 15.6% (95% CI 13.5, 17.2), respectively. The prevalence of delay was 5–8% higher among hypertension-exposed children compared to unexposed children.

**Table 2:** Estimated prevalence of developmental delays in children at 36 months.

	Overall	Hypertensive Status	
		HDP+	HDP-
	Prevalence	Prevalence	Prevalence
	(95% CI)	(95% CI)	(95% CI)
Any delay	32.0	39.8	31.4
	(29.7, 34.4)	(31.5, 48.8)	(29.0, 33.8)
Motor delay	23.5	30.1	23.0
	(21.5, 25.7)	(22.5, 38.9)	(20.8, 25.2)
Cognitive delay	15.2	20.2	15.2
	(13.5, 17.2)	(13.9, 28.3)	(13.5, 17.2)

HDP = hypertensive disorders in pregnancy. CI = confidence interval.

Results from logistic regression modelling are displayed in Table 3. We did not find evidence of modification by child sex, with all p-values for interaction >0.05 and similar effect sizes across males and females (results available upon request). RRs between HDP and developmental delay were attenuated modestly after adjusting for confounders (ARR1), and minimally after additional adjustment for mediators (ARR2). Effect size estimates were invariably greater than 1, indicating an elevated risk of developmental delay in hypertension-exposed children relative to unexposed children, but were not statistically significant. After controlling for maternal and obstetric characteristics, the RRs quantifying the fully adjusted effect of HDP on child development were 1.19 (95% CI 0.92, 1.53) for any delay, 1.18 (95% CI 0.86, 1.61) for motor delay, and 1.24 (95% CI 0.83, 1.85) for cognitive delay.

**Table 3:** Staged logistic regression modelling of the association between hypertensive disorders in pregnancy and developmental delays at 36 months.

	Crude RR	ARR1	ARR2
	(95% CI)	(95% CI)	(95% CI)
Any delay	n = 1539	n = 1451	n = 1441
HDP-	1.00 (Reference)	1.00 (Reference)	1.00 (Reference)
HDP+	1.27	1.21	1.19
	(1.01, 1.60)	(0.94, 1.55)	(0.92, 1.53)
Motor delay	n = 1547	n = 1459	n = 1449
HDP-	1.00 (Reference)	1.00 (Reference)	1.00 (Reference)
HDP+	1.31	1.20	1.18
	(0.98, 1.74)	(0.89, 1.63)	(0.86, 1.61)
Cognitive delay	n = 1542	n = 1453	n = 1443
HDP-	1.00 (Reference)	1.00 (Reference)	1.00 (Reference)
HDP+	1.32	1.28	1.24
	(0.91, 1.92)	(0.86, 1.89)	(0.83, 1.85)

HDP = hypertensive disorders in pregnancy. RR = risk ratio. CI = confidence interval. ARR1 = adjusted for confounders (sociodemographic vulnerability, maternal age, pre-pregnancy overweight/obesity, prenatal depression, sex). ARR2 = adjusted for confounders and mediators (postpartum depression, gestational age).

Sensitivity analysis restricting to pre-eclampsia/eclampsia cases is presented in S3 Table. RRs were consistently slightly higher than those from our main analysis (using composite HDP), indicating an elevated risk of developmental delay in pre-eclampsia/eclampsia-exposed children relative to unexposed children, but were not statistically significant. The aRRs quantifying the fully adjusted effect of pre-eclampsia/eclampsia on child development were 1.27 (95% CI 0.98, 1.64) for any delay, 1.27 (95% CI 0.92, 1.74) for motor delay, and 1.29 (95% CI 0.85, 1.95) for cognitive delay. Sensitivity analysis restricting to children born full-term is presented in S4 Table. RRs did not materially differ from those in our main analysis (where we adjusted for gestational age), though precision was slightly lower as evidenced by wider 95% CIs.

## Discussion

In this prospective community-based cohort study, we did not find a statistically significant association between HDP and any, motor, or cognitive delay in children at 36 months. CIs suggested similar or slightly elevated risk of any delay in children exposed to HDP compared to unexposed peers, but crossed the null value in crude and adjusted models. The magnitude of point estimates between HDP and developmental delay persisted after controlling for sociodemographic, maternal, obstetric, and mental health covariates decreasing slightly with each adjustment, and with restriction to full-term births. Moreover, we did not find evidence of modification by child sex.

Few studies have examined the association between HDP and child development before 3 years using a large community-based cohort design. Using a Canadian sample of 129 mothers with severe preeclampsia and 140 normotensive age-, race-, and parity-matched controls, Warshafsky et al. reported a greater proportion of exposed infants had suboptimal development (i.e., failed ≥1 ASQ domain) each year in the first 5 years of life [19]. Researchers in Finland examined the relationship between HDP types and an ordinal score of ASQ domain failure at a mean of age 42.1 months in 2504 mother-child dyads, and reported a significant association between preeclampsia and increasingly severe/pervasive delay after confounder adjustments (AOR 1.52, 95% CI 1.04, 2.23), but not gestational hypertension (AOR 0.82, 95% CI 0.53, 1.29) or chronic hypertension (AOR 0.89, 95% CI 0.93, 1.62) [36]. Chen et al. followed 4031 mother-infant dyads in China to determine the impact of HDP types on domain-specific development scores and odds of delay at 6 months, as measured by blinded pediatricians using the Gesell Development Schedules [37]. They concluded that chronic hypertension was associated with higher odds of delay on all domains except for gross motor, gestational hypertension was associated with significantly lower mean scores on the social behavior domain only, and preeclampsia was not significantly associated with differences in scores or odds of delay.

Adding to this literature, our results suggest there is no statistically significant effect of HDP on developmental delay at 36 months. However, heeding recent calls to broaden scientific interpretations beyond significance thresholds [38], it is noteworthy that although our adjusted effect sizes were small (RRs≈1.20), they were consistent and in the direction of elevated risk for any, motor, and cognitive delay following prenatal hypertension exposure after accounting for all pertinent covariates. Moreover, RRs increased in magnitude when we restricted to pre-eclampsia/eclampsia cases only, which is consistent with the understanding that these disorders represent the most serious manifestations of HDP. Turning attention to the CIs, our data appear to be compatible with effect sizes ranging from a small protective or null effect as indicated by the lower bound values (RR≈0.85), which is countered by a larger detrimental effect as indicated by the upper bound values (RR≈1.6) of HDP on risk of delay. From a clinical perspective, a null association warrants no change to clinical practice, whereas an elevated risk of developmental delay in children exposed to HDP in utero evident by age 3 suggests that closer neurodevelopmental assessment of these children during the early years may be beneficial. Such assessment could aid with identifying children experiencing or at-risk of delays, and facilitating access to supports before school entry when they are poised to have the largest positive impact on children’s developmental trajectory [39].

There is also compelling evidence for the biological mechanisms by which hypertension during gestation, and particularly pre-eclampsia, can impede neurodevelopment. Hypertension-related constriction of blood vessels introduces hypoxia in the placental environment, and research in animal models has shown that acute and chronic hypoxic insults can lead to permanent changes in brain structure [9]. On a cellular level, neurons are among the most sensitive to decreases in oxygen which can impede normal cellular maturation and, in severe cases, lead to apoptosis [40]. Blood vessel formation may also be impaired from hypertension, with recent studies implicating the VEGF family of proteins which promote angiogenesis. Data in both animals and humans has shown lower concentrations of certain VEGF proteins (namely placental growth factor) and higher concentrations of sFlt-1 receptors that block VEGF protein activity in maternal plasma and cord blood of preeclamptic subjects, suggesting a disruption in blood vessel development [41]. Pro-inflammatory cytokines have been found in elevated levels in the plasma of pregnant hypertensive women [42, 43], and findings from animal models suggest that cytokine-mediated inflammation may affect the fetal brain by directly injuring neurons or triggering damage to surrounding tissue [44]. Lastly, preeclampsia appears to be associated with altered transport and metabolism of omega-3 fatty acids in placental tissue, as well as reduced levels of fatty acids in maternal plasma and cord blood after accounting for dietary intake [45]. Limited quantity of omega-3 fatty acids to the fetus is problematic given the critical role these molecules play in developing the structure and function of nervous system cells [46]. The functional implications of these physiologic differences between hypertensive and normotensive pregnancies is not entirely clear, and represents an active area of investigation. Nonetheless, researchers have identified several plausible pathways whereby hypertension-induced changes to the maternal-placental-fetal unit can adversely impact fetal brain development, which may lead to observable differences in developmental scores and/or risk of delay in early childhood and beyond.

A major strength of this analysis is our use of a community-based sample of over 1500 mother-infant dyads. Much of the existing research on this topic has used small, clinic-based samples of preterm or small infants, from which findings are not necessarily transferable to the general neonatal population. The ASQ-3 and its normative cut-offs used to measure developmental delay are commonly used by clinicians and educators to identify children at risk, lending to the applicability of our results for community settings. To maximize transparency, we documented our assumptions about the relationship between variables in a DAG and used this to guide our staged modelling. This approach enabled us to estimate the effect of HDP on developmental delay without over-adjusting for covariates not hypothesized to be on a causal pathway between the two. Such over-adjustments can degrade precision and/or open biasing pathways that distort the estimated association [35, 47]. Given emerging research on the bi-directional relationship between HDP and depression [48, 49] and established research on the impact of maternal mental health on toddler development [50], we included maternal depression covariates in our analysis expecting it to positively confound (prenatal depression) or lie on the causal pathway (postpartum depression) between HDP and child development. Importantly, maternal mental health has not been integrated into previous studies addressing this topic. Our analysis suggests that maternal depression plays only a minor role in influencing the effect of HDP on child development, as evidenced by minimal attenuations in the size of RRs when mental health variables were included in the models. In light of evidence for sex-dependent responses to insults in utero [51], we assessed whether the estimated associations differed across child sex; the absence of sex modification we observed is consistent with the limited previous research that included a similar assessment [22].

Findings should be considered in light of several limitations. Despite our modest sample size, low cell counts for hypertensive women precluded us from separately analyzing individual ASQ-3 domains or from assessing for effect modification by socioeconomic status [52]. A larger sample size may have afforded us the opportunity to explore these elements, as well as improved the precision of our results; however, our sample size is quite large when considered in light of existing studies analyzing prenatal determinants of child development (often <500 participants) [50, 53, 54]. This study did not attempt to broadly determine risk factors for developmental delay; our focus on a hypothesis-driven analysis, using a DAG, was intended to solely capture the potential effect of HDP on delay in children at 36 months. Other known determinants of cognitive and motor delay in the early years, such as parental education, income, childcare quality, and parenting style [55], should always be considered when identifying intervention opportunities. Data on the timing and severity of HDP were unavailable, and thus our measurement of HDP is heterogeneous and precludes us from elucidating the relative effects of early versus late-onset, or mild versus severe HDP on developmental outcomes. Detailed information on maternal medications were also unavailable; certain medications, such as anti-depressants [56–59], are associated with greater risk of HDP and may adversely affect child development and could therefore be a source of unmeasured confounding. We could only include roughly half of the AOF cohort in this analysis. Of the original 3388 participants, 1993 completed the 3-year questionnaire and 1553 had complete and valid data to answer our research question. Participants in our analysis were somewhat more likely to be older, primiparous, well educated, and have sufficient income compared to both the full AOF sample and the general Alberta population (see S2 Table). These differences may explain the 8% prevalence of HDP observed in the sample, which is marginally higher than the 6% prevalence observed at the provincial level [1], given that older maternal age and primiparity are risk factors for HDP [60]. This is unlikely to introduce selection bias, given that a slight over-representation of hypertensive women is not expected to differ according to developmental delay groups. Rather, generalization of our findings to vulnerable populations should be done with caution.

Given the lack of statistical significance observed (using the traditional categorization with 5% significance level), it is possible that HDP truly has no effect on child development or that unmeasured confounding is responsible for the differences that we and others have reported. When considered more broadly with respect to the range of plausible values for this relationship based on our data, and compatibility with the epidemiologic and biological evidence to date, our findings cautiously add to the plausibility that HDP may adversely influence child development at 36 months, although the relationship is still uncertain. Future epidemiologic research is warranted to investigate this relationship, and we would recommend leveraging large samples or meta-analysis, quantifying the direct (biological) effect, and operationalizing delay with a clinically meaningful threshold. Optimal development during childhood provides the foundation for healthy social and physical functioning into adolescence and adulthood. A better understanding of the role of HDP on children’s neurodevelopment can inform monitoring of developmental progress, communication with families, and early intervention following prenatal hypertension exposure, as well as upstream efforts to prevent HDP and its negative sequelae on maternal and child health.

## Conclusion

Although not statistically significant, our results suggest that children exposed to HDP may have either similar or slightly elevated risk of any, motor, and cognitive delay at 36 months after controlling for maternal and obstetric characteristics. Importantly, the direction of association we observed aligns with accumulating evidence on biological mechanisms through which hypertension in utero adversely impacts fetal brain development. Additional research would be valuable to clarify the effect of HDP on cognitive and motor delay. Knowledge that HDP may pose additional risks for developmental delay allows for the identification of at-risk children in infancy, thus enabling prompt intervention and additional supports to be deployed in early childhood when they are at peak availability and effectiveness.

## Supporting information

S1 Checklist STROBE statement—checklist of items that should be included in reports of cohort studies. (DOC)

S1 Table Extent of missing data for covariate and outcome variables. (DOCX)

S2 Table Comparison between baseline characteristics of the analytic sample, the full All Our Families sample, and the Alberta population. (DOCX)

S3 Table Staged logistic regression modelling of the association between pre-eclampsia/eclampsia and developmental delays at 36 months. (DOCX)

S4 Table Staged logistic regression modelling of the association between hypertensive disorders of pregnancy and developmental delays at 36 months among children born full-term. (DOCX)

## References

[1] Roberts CL, Ford JB, Algert CS, Antonsen S, Chalmers J, Cnattingius S, et al. Population-based trends in pregnancy hypertension and pre-eclampsia: an international comparative study. BMJ Open. 2011;2: e00101. doi: 10.1136/bmjopen-2011-000101 22021762 PMC3191437

[2] Zhang J, Meikle S, Trumble A. Severe Maternal Morbidity Associated with Hypertensive Disorders in Pregnancy in the United States. Hypertens Pregnancy. 2003;22: 203–212. doi: 10.1081/PRG-120021066 12909005

[3] Roberts LM, Davis GK, Homer CSE. Pregnancy with gestational hypertension or preeclampsia: A qualitative exploration of women’s experiences. Midwifery. 2017;46: 17–23. doi: 10.1016/j.midw.2017.01.004 28110162

[4] Ananth C V., Vintzileos AM. Maternal-fetal conditions necessitating a medical intervention resulting in preterm birth. Am J Obstet Gynecol. 2006;195: 1557–1563. doi: 10.1016/j.ajog.2006.05.021 17014813

[5] Ødegård RA, Vatten LJ, Nilsen ST, Salvesen KÅ, Austgulen R. Preeclampsia and fetal growth. Obstet Gynecol. 2000;96: 950–955. doi: 10.1016/S0029-7844(00)01040-1 11084184

[6] Allen VM, Joseph K, Murphy KE, Magee LA, Ohlsson A. The effect of hypertensive disorders in pregnancy on small for gestational age and stillbirth: a population based study. BMC Pregnancy Childbirth. 2004;4: 17. doi: 10.1186/1471-2393-4-17 15298717 PMC515178

[7] Basso O, Rasmussen S, Weinberg CR, Wilcox AJ, Irgens LM, Skjaerven R. Trends in fetal and infant survival following preeclampsia. J Am Med Assoc. 2006;296: 1357–1362. doi: 10.1001/jama.296.11.1357 16985227

[8] Maher GM, O’Keeffe GW, Kearney PM, Kenny LC, Dinan TG, Mattsson M, et al. Association of hypertensive disorders of pregnancy with risk of neurodevelopmental disorders in offspring a systematic review and meta-analysis. JAMA Psychiatry. 2018;75: 809–819. doi: 10.1001/jamapsychiatry.2018.0854 29874359 PMC6143097

[9] Rees S, Harding R, Walker D. An adverse intrauterine environment: implications for injury and altered development of the brain. Int J Dev Neurosci. 2008;26: 3–11. doi: 10.1016/j.ijdevneu.2007.08.020 17981423

[10] Mann JR, McDermott S, Griffith MI, Hardin J, Gregg A. Uncovering the complex relationship between pre-eclampsia, preterm birth and cerebral palsy. Paediatr Perinat Epidemiol. 2011;25: 100–110. doi: 10.1111/j.1365-3016.2010.01157.x 21281322

[11] Wilcox AJ, Weinberg CR, Basso O. On the pitfalls of adjusting for gestational age at birth. Am J Epidemiol. 2011;174: 1062–1068. doi: 10.1093/aje/kwr230 21946386 PMC3243938

[12] Sun B, Moster D, Harmon Q, Wilcox A. Association of Preeclampsia in Term Births With Neurodevelopmental Disorders in Offspring. JAMA Psychiatry. 2020; Epub ahead of print. doi: 10.1001/jamapsychiatry.2020.0306 32236510 PMC7113825

[13] Many A, Fattal A, Leitner Y, Kupferminc MJJ, Harel S, Jaffa A. Neurodevelopmental and cognitive assessment of children born growth restricted to mothers with and without preeclampsia. Hypertens Pregnancy. 2003;22: 25–29. doi: 10.1081/PRG-120016791 12648440

[14] Cheng SW, Chou HC, Tsou KI, Fang LJ, Tsao PN. Delivery before 32 weeks of gestation for maternal pre-eclampsia: Neonatal outcome and 2-year developmental outcome. Early Hum Dev. 2004;76: 39–46. doi: 10.1016/j.earlhumdev.2003.10.004 14729161

[15] Silveira RC, Procianoy RS, Koch MS, Benjamin ACW, Schlindwein CF. Growth and neurodevelopment outcome of very low birth weight infants delivered by preeclamptic mothers. Acta Paediatr. 2007;96: 1738–1742. doi: 10.1111/j.1651-2227.2007.00552.x 17953726

[16] McCowan LME, Pryor J, Harding JE. Perinatal predictors of neurodevelopmental outcome in small-for-gestational-age children at 18 months of age. Am J Obstet Gynecol. 2002;186: 1069–1075. doi: 10.1067/mob.2002.122292 12015539

[17] Avorgbedor F, Silva S, Merwin E, Blumenthal JA, Holditch-Davis D. Health, Physical Growth, and Neurodevelopmental Outcomes in Preterm Infants of Women With Hypertensive Disorders of Pregnancy. J Obstet Gynecol Neonatal Nurs. 2019;48: 69–77. doi: 10.1016/j.jogn.2018.10.003 30502314 PMC6321773

[18] Schlapbach LJ, Ersch J, Adams M, Bernet V, Bucher HU, Latal B. Impact of chorioamnionitis and preeclampsia on neurodevelopmental outcome in preterm infants below 32 weeks gestational age. Acta Paediatr. 2010;99: 1504–1509. doi: 10.1111/j.1651-2227.2010.01861.x 20456275

[19] Warshafsky C, Pudwell J, Walker M, Wen S-W, Smith GN. Prospective assessment of neurodevelopment in children following a pregnancy complicated by severe pre-eclampsia. BMJ Open. 2016;6: e010884. doi: 10.1136/bmjopen-2015-010884 27388354 PMC4947739

[20] Heikura U, Hartikainen AL, Nordstrom T, Pouta A, Taanila A, Jarvelin MR. Maternal hypertensive disorders during pregnancy and mild cognitive limitations in the offspring. Paediatr Perinat Epidemiol. 2013;27: 188–198. doi: 10.1111/ppe.12028 23374064

[21] Whitehouse AJO, Robinson M, Newnham JP, Pennell CE. Do hypertensive diseases of pregnancy disrupt neurocognitive development in offspring? Paediatr Perinat Epidemiol. 2012;26: 101–108. doi: 10.1111/j.1365-3016.2011.01257.x 22324495

[22] Grace T, Bulsara M, Pennell C, Hands B. Maternal hypertensive diseases negatively affect offspring motor development. Pregnancy Hypertens. 2014;4: 209–214. doi: 10.1016/j.preghy.2014.04.003 26104607

[23] McDonald SW, Lyon AW, Benzies KM, McNeil DA, Lye SJ, Dolan SM, et al. The All Our Babies pregnancy cohort: design, methods, and participant characteristics. BMC Pregnancy Childbirth. 2013;13: S2. doi: 10.1186/1471-2393-13-S1-S2 23445747 PMC3561154

[24] Hutcheon JA, Lisonkova S, Joseph KS. Epidemiology of pre-eclampsia and the other hypertensive disorders of pregnancy. Best Pract Res Clin Obstet Gynaecol. 2011;25: 391–403. doi: 10.1016/j.bpobgyn.2011.01.006 21333604

[25] Squires J, Twombly E, Bricker D, Potter L. ASQ-3 User’s Guide. Baltimore, MD: Paul H. Brookes Publishing Co., Inc; 2009.

[26] Velikonja T, Edbrooke-Childs J, Calderon A, Sleed M, Brown A, Deighton J. The psychometric properties of the Ages & Stages Questionnaires for ages 2–2.5: a systematic review. Child Care Health Dev. 2017;43: 1–17. doi: 10.1111/cch.12397 27554865

[27] Schonhaut L, Armijo I, Schönstedt M, Alvarez J, Cordero M. Validity of the ages and stages questionnaires in term and preterm infants. Pediatrics. 2013;131. doi: 10.1542/peds.2012-3313 23629619

[28] Limbos MM, Joyce DP. Comparison of the ASQ and PEDS in screening for developmental delay in children presenting for primary care. J Dev Behav Pediatr. 2011;32: 499–511. doi: 10.1097/DBP.0b013e31822552e9 21760526

[29] Shavers VL. Measurement of socioeconomic status in health disparities research. J Natl Med Assoc. 2007;99: 1013–23. doi: 10.1037/e518882014-001 17913111 PMC2575866

[30] Gibson J, McKenzie-Mcharg K, Shakespeare J, Price J, Gray R. A systematic review of studies validating the Edinburgh Postnatal Depression Scale in antepartum and postpartum women. Acta Psychiatr Scand. 2009;119: 350–364. doi: 10.1111/j.1600-0447.2009.01363.x 19298573

[31] Cox JL, Holden JM, Sagovsky R. Detection of postnatal depression: Development of the 10-item Edinburgh Postnatal Depression Scale. Br J Psychiatry. 1987;150: 782–786. doi: 10.1192/bjp.150.6.782 3651732

[32] Norton EC, Miller MM, Kleinman LC. Computing adjusted risk ratios and risk differences in Stata. Stata J. 2013;13: 492–509. doi: 10.1177/1536867x1301300304

[33] Rothman KJ, Greenland S, Lash TL. Modern Epidemiology. 3rd ed. Philadelphia, PA: Lippincott Williams & Wilkins; 2008.

[34] Harrell F. Regression Modeling Strategies With Applications to Linear Models, Logistic and Ordinal Regression, and Survival Analysis. 2nd ed. Springer Series in Statistics. Springer Science & Business Media; 2015.

[35] Ananth C V, Schisterman EF. Confounding, Causality and Confusion: The Role of Intermediate Variables in Interpreting Observational Studies in Obstetrics. Am J Obstet Gynecol. 2017;217: 167–175. doi: 10.1016/j.ajog.2017.04.016 28427805 PMC5545051

[36] Girchenko P, Tuovinen S, Lahti-Pulkkinen M, Lahti J, Savolainen K, Heinonen K, et al. Maternal early pregnancy obesity and related pregnancy and pre-pregnancy disorders: Associations with child developmental milestones in the prospective PREDO Study. Int J Obes. 2018;42: 995–1007. doi: 10.1038/s41366-018-0061-x 29686379

[37] Chen Z, Li R, Liu H, Duan J, Yao C, Yang R, et al. Impact of maternal hypertensive disorders on offspring’s neurodevelopment: a longitudinal prospective cohort study in China. Pediatr Res. 2020; 1–8. doi: 10.1038/s41390-020-0794-9 32018276

[38] Amrhein V, Mcshane B, Greenland S. Retire statistical significance. Nature. 2019;567: 305–307. doi: 10.1038/d41586-019-00857-9 30894741

[39] Doyle A, Harmon C, Heckman J, Tremblay R. Investing in Early Human Development: Timing and Economic Efficiency. Econ Hum Biol. 2009;7: 1–6. doi: 10.1016/j.ehb.2009.01.002 19213617 PMC2929559

[40] Nalivaeva NN, Turner AJ, Zhuravin IA. Role of prenatal hypoxia in brain development, cognitive functions, and neurodegeneration. Front Neurosci. 2018;12: 1–21. doi: 10.3389/fnins.2018.00001 30510498 PMC6254649

[41] Lara E, Acurio J, Leon J, Penny J, Torres-Vergara P, Escudero C. Are the cognitive alterations present in children born from preeclamptic pregnancies the result of impaired angiogenesis? Focus on the potential role of the VEGF family. Front Physiol. 2018;9: 1–10. doi: 10.3389/fphys.2018.00001 30487752 PMC6246680

[42] Ferguson KK, Meeker JD, McElrath TF, Mukherjee B, Cantonwine DE. Repeated measures of inflammation and oxidative stress biomarkers in preeclamptic and normotensive pregnancies. Am J Obstet Gynecol. 2017;216: 527.e1–527.e9. doi: 10.1016/j.ajog.2016.12.174 28043842 PMC5420472

[43] Tangerås LH, Austdal M, Skråstad RB, Salvesen KA, Austgulen R, Bathen TF, et al. Distinct First Trimester Cytokine Profiles for Gestational Hypertension and Preeclampsia. Arterioscler Thromb Vasc Biol. 2015;35: 2478–2485. doi: 10.1161/ATVBAHA.115.305817 26404486

[44] Burd I, Balakrishnan B, Kannan S. Models of Fetal Brain Injury, Intrauterine Inflammation, and Preterm Birth. Am J Reprod Immunol. 2012;67: 287–294. doi: 10.1111/j.1600-0897.2012.01110.x 22380481

[45] Wadhwani N, Patil V, Pisal H, Joshi A, Mehendale S, Gupte S, et al. Altered maternal proportions of long chain polyunsaturated fatty acids and their transport leads to disturbed fetal stores in preeclampsia. Prostaglandins Leukot Essent Fat Acids. 2014;91: 21–30. doi: 10.1016/j.plefa.2014.05.006 24928794

[46] Devarshi PP, Grant RW, Ikonte CJ, Hazels Mitmesser S. Maternal Omega-3 Nutrition, Placental Transfer and Fetal Brain Development in Gestational Diabetes and Preeclampsia. Nutrients. 2019;11: 1107. doi: 10.3390/nu11051107 31109059 PMC6567027

[47] Kramer MS, Zhang X, Dahhou M, Yang S, Martin RM, Oken E, et al. Does fetal growth restriction cause later obesity? Pitfalls in analyzing causal mediators as confounders. Am J Epidemiol. 2017;185: 585–590. doi: 10.1093/aje/kww109 28338874 PMC5860505

[48] Horsley KJ, Tomfohr-Madsen LM, Ditto B, Tough SC. Hypertensive Disorders of Pregnancy and Symptoms of Depression and Anxiety as Related to Gestational Age at Birth: Findings From the All Our Families Study. Psychosom Med. 2019;81: 458–463. doi: 10.1097/PSY.0000000000000695 30985405

[49] Shay M, MacKinnon AL, Metcalfe A, Giesbrecht G, Campbell T, Nerenberg K, et al. Depressed mood and anxiety as risk factors for hypertensive disorders of pregnancy: A systematic review and meta-analysis. Psychol Med. 2020;50: 2128–2140. doi: 10.1017/S0033291720003062 32912348

[50] Kingston D, McDonald S, Austin MP, Tough S. Association between prenatal and postnatal psychological distress and toddler cognitive development: A systematic review. PLoS One. 2015;10: 1–16. doi: 10.1371/journal.pone.0126929 25996151 PMC4440779

[51] Clifton VL. Review: Sex and the Human Placenta: Mediating Differential Strategies of Fetal Growth and Survival. Placenta. 2010;24: S33–S39. doi: 10.1016/j.placenta.2009.11.010 20004469

[52] Lawson G, Hook C, Farah M. A meta-analysis of the relationship between socioeconomic status and executive function performance among children. Dev Sci. 2018;21: 139–148. doi: 10.1111/desc.12529 28557154 PMC5821589

[53] Sharapova SR, Phillips E, Sirocco K, Kaminski JW, Leeb RT, Rolle I. Effects of prenatal marijuana exposure on neuropsychological outcomes in children aged 1–11 years: A systematic review. Paediatr Perinat Epidemiol. 2018;32: 512–532. doi: 10.1111/ppe.12505 30335203 PMC6261687

[54] Adane AA, Mishra GD, Tooth LR. Diabetes in pregnancy and childhood cognitive development: A systematic review. Pediatrics. 2016;137. doi: 10.1542/peds.2015-4234 27244820

[55] Maggi S, Irwin LJ, Siddiqi A, Hertzman C. The social determinants of early child development: An overview. J Paediatr Child Health. 2010;46: 627–635. doi: 10.1111/j.1440-1754.2010.01817.x 20796183

[56] De Ocampo MPG, Araneta MRG, Macera CA, Alcaraz JE, Moore TR, Chambers CD. Risk of gestational hypertension and preeclampsia in women who discontinued or continued antidepressant medication use during pregnancy. Arch Womens Ment Health. 2016;19: 1051–1061. doi: 10.1007/s00737-016-0655-z 27558246

[57] Bernard N, Forest JC, Tarabulsy GM, Bujold E, Bouvier D, Giguère Y. Use of antidepressants and anxiolytics in early pregnancy and the risk of preeclampsia and gestational hypertension: A prospective study. BMC Pregnancy Childbirth. 2019;19: 1–9. doi: 10.1186/s12884-018-2145-y 31039756 PMC6492434

[58] Prady SL, Hanlon I, Fraser LK, Mikocka-Walus A. A systematic review of maternal antidepressant use in pregnancy and short- and long-term offspring’s outcomes. Arch Womens Ment Health. 2018;21: 127–140. doi: 10.1007/s00737-017-0780-3 29027013 PMC5856864

[59] Lupattelli A, Wood M, Ystrom E, Skurtveit S, Handal M, Nordeng H. Effect of Time-Dependent Selective Serotonin Reuptake Inhibitor Antidepressants During Pregnancy on Behavioral, Emotional, and Social Development in Preschool-Aged Children. J Am Acad Child Adolesc Psychiatry. 2018;57: 200–208. doi: 10.1016/j.jaac.2017.12.010 29496129 PMC5843872

[60] Duckitt K, Harrington D. Risk factors for pre-eclampsia at antenatal booking: systematic review of controlled studies. BMJ. 2005;330: 565. doi: 10.1136/bmj.38380.674340.E0 15743856 PMC554027

