# Supplementary material for: Hypertensive disorders in pregnancy and child development at 36 months in the All Our Families prospective cohort study
Source: PLoS One. 2021 Dec 1;16(12):e0260590. doi: 10.1371/journal.pone.0260590 (PMC8635344; doi:10.1371/journal.pone.0260590)
Supplement: S1 Table — (DOCX) [file pone.0260590.s002.docx]

**S1 Table.** Extent of missing data for covariate and outcome variables

|  | Overall |
| --- | --- |
| Variable | N=1554  n (%) |
| Covariates |  |
| Maternal age | 42 (2.7) |
| Sociodemographic vulnerability | 20 (1.3) |
| Marital status | 2 (0.1) |
| Home ownership | 0 (0) |
| Primary language | 1 (0.1) |
| Annual income | 56 (3.6) |
| Education | 3 (0.2) |
| Pre-pregnancy BMI | 18 (1.2) |
| Parity | 12 (0.8) |
| Perinatal mental health |  |
| Prenatal depression | 15 (1.0) |
| Postpartum depression | 32 (2.1) |
| Mode of delivery | 25 (1.6) |
| Gestational age at birth | 0 (0) |
| Child sex | 0 (0) |
| Outcome |  |
| Domains |  |
| Gross motor | 2 (0.1) |
| Fine motor | 6 (0.4) |
| Communication | 3 (0.2) |
| Problem solving | 8 (0.5) |
| Personal-social | 5 (0.3) |
| Any delay | 15 (1.0) |
| Motor delay | 7 (0.5) |
| Cognitive delay | 12 (0.8) |

BMI=body mass index. Approach to determining missing data for the any, motor, and cognitive delay variables: if a child was delayed on 0 pertinent domains but missing data for 1 pertinent domain, we were unable to definitively classify their developmental status. For example, if a child had normal personal-social and communication domain scores but was missing the problem-solving score, we conservatively classified that child as ‘missing’ for the cognitive delay variable given that we cannot say with certainty whether they experienced a delay in *at least 1* pertinent domain. Differences in the proportion of missing data were not differential across hypertensive status.
