# Supplementary material for: Hypertensive disorders in pregnancy and child development at 36 months in the All Our Families prospective cohort study
Source: PLoS One. 2021 Dec 1;16(12):e0260590. doi: 10.1371/journal.pone.0260590 (PMC8635344; doi:10.1371/journal.pone.0260590)
Supplement: S2 Table — (DOCX) [file pone.0260590.s003.docx]

**S2 Table.** Comparison between baseline characteristics of the analytic sample, the full All Our Families sample, and the Alberta population

| Characteristic | Analysis  N=1554  % | AOF  N=3388  % | Alberta  N=NA  % |
| --- | --- | --- | --- |
| Maternal age at delivery ≥35 years | 26.1 | 24.1 | 15.6 |
| Annual income >$40,000 | 94.3 | 92.3 | 77.8 |
| Post-secondary completed | 80.0 | 76.3 | 69.5 |
| Pre-pregnancy BMI, mean | 24.4 | 24.3 | 24.4 |
| Primiparous | 50.6 | 48.9 | 46.0 |
| Prenatal depression score ≥13 | 9.9 | 12.0 | NA |
| Postpartum depression score ≥13 | 4.9 | 5.1 | 6.5 |
| Cesarean delivery | 24.9 | 24.5 | 27.3 |
| Preterm birth | 5.7 | 7.3 | 6.3 |

NA=not available. All Our Families sample data and Alberta population data from McDonald et al., 2013.
