# Supplementary material for: Hypertensive disorders in pregnancy and child development at 36 months in the All Our Families prospective cohort study
Source: PLoS One. 2021 Dec 1;16(12):e0260590. doi: 10.1371/journal.pone.0260590 (PMC8635344; doi:10.1371/journal.pone.0260590)
Supplement: S3 Table — (DOCX) [file pone.0260590.s004.docx]

**S3 Table.** Staged logistic regression modelling of the association between pre-eclampsia/eclampsia and developmental delays at 36 months

|  | Prev. of delay  (95% CI) | Crude RR  (95% CI) | ARR1  (95% CI) | ARR2  (95% CI) |
| --- | --- | --- | --- | --- |
| Any delay | n=1539 |  | n=1451 | n=1441 |
| PE- | 31.3  (28.9, 33.7) | 1.00 (Reference) | 1.00 (Reference) | 1.00 (Reference) |
| PE + | 42.2  (33.2, 51.8) | **1.35**  **(1.07, 1.70)** | **1.29**  **(1.00, 1.65)** | 1.27  (0.98, 1.64) |
| Motor delay | n=1547 |  | n=1459 | n=1449 |
| PE- | 22.9  (20.8, 25.1) | 1.00 (Reference) | 1.00 (Reference) | 1.00 (Reference) |
| PE + | 32.1  (23.9, 41.6) | **1.40**  **(1.05, 1.87)** | 1.29  (0.94, 1.76) | 1.27  (0.92, 1.74) |
| Cognitive delay | n=1542 |  | n=1453 | n=1443 |
| PE- | 15.2  (13.5, 17.2) | 1.00 (Reference) | 1.00 (Reference) | 1.00 (Reference) |
| PE + | 20.9  (14.2, 29.7) | 1.37  (0.94, 2.02) | 1.32  (0.88, 1.99) | 1.29  (0.85, 1.95) |

PE=pre-eclampsia or eclampsia. RR=risk ratio. CI=confidence interval. ARR1=adjusted for confounders (sociodemographic vulnerability, maternal age, pre-pregnancy overweight/obesity, prenatal depression, sex). ARR2=adjusted for confounders and mediators (postpartum depression, gestational age).
