# Supplementary material for: Hypertensive disorders in pregnancy and child development at 36 months in the All Our Families prospective cohort study
Source: PLoS One. 2021 Dec 1;16(12):e0260590. doi: 10.1371/journal.pone.0260590 (PMC8635344; doi:10.1371/journal.pone.0260590)
Supplement: S4 Table — (DOCX) [file pone.0260590.s005.docx]

**S4 Table.** Staged logistic regression modelling of the association between hypertensive disorders of pregnancy and developmental delays at 36 months among children born full-term

|  | Prev. of delay  (95% CI) | Crude RR  (95% CI) | ARR1  (95% CI) | ARR2  (95% CI) |
| --- | --- | --- | --- | --- |
| Any delay | n=1450 |  | n=1373 | n=1365 |
| HDP- | 30.8  (28.4, 33.3) | 1.00 (Reference) | 1.00 (Reference) | 1.00 (Reference) |
| HDP+ | 38.4  (29.2, 48.5) | 1.25  (0.96, 1.62) | 1.22  (0.93, 1.60) | 1.21  (0.93, 1.60) |
| Motor delay | n=1459 |  | n=1382 | n=1374 |
| HDP- | 22.4  (20.3, 24.7) | 1.00 (Reference) | 1.00 (Reference) | 1.00 (Reference) |
| HDP+ | 29.3  (21.1, 39.2) | 1.31  (0.95, 1.80) | 1.25  (0.89, 1.74) | 1.25  (0.89, 1.74) |
| Cognitive delay | n=1453 |  | n=1375 | n=1367 |
| HDP- | 14.9  (13.1, 16.9) | 1.00 (Reference) | 1.00 (Reference) | 1.00 (Reference) |
| HDP+ | 20.0  (13.2, 29.2) | 1.34  (0.89, 2.02) | 1.30  (0.84, 2.01) | 1.31  (0.85, 2.01) |

HDP=hypertensive disorders in pregnancy. RR=risk ratio. CI=confidence interval. ARR1=adjusted for confounders (sociodemographic vulnerability, maternal age, pre-pregnancy overweight/obesity, prenatal depression, sex). ARR2=adjusted for confounders and mediators (postpartum depression).
